# Pragmatic approaches to mitigating missing data and research priorities to assess the effectiveness of interventions

**DOI:** 10.1186/1745-6215-16-S2-P238

**Published:** 2015-11-16

**Authors:** Anna Kearney, Anne Daykin, Ali Heawood, Athene Lane, Jane Blazeby, Mike Clarke, Paula Williamson, Carrol Gamble

**Affiliations:** 1 University of Liverpool, Liverpool, UK; 2 University of Bristol, Bristol, UK; 3 Queen's University, Belfast, UK

## Background

Identifying interventions to minimise missing data was the third highest research priority in a Delphi survey of the Directors of UK Clinical Trial Units (CTUs). However, a Cochrane Methodology Review of nested randomised studies of missing data interventions shows a substantial evidence gap, with all but one of the eligible studies targeting questionnaire response rates.

Research is needed to identify strategies that effectively address the broader causes of missing data, including minimising withdrawals, ensuring clinical staff capture vital outcome measurements and improving patient attendance at clinic visits.

## Methods

Chief Investigators of HTA funded trials and the CTUs were surveyed to identify frequently used interventions, their perceived effectiveness and any implementation problems.

A subsequent two round Delphi survey was conducted with CTUs to gain consensus around which interventions should undergo further research to formally evaluate their effectiveness.

## Results

We discuss the frequency and range of pragmatic interventions implemented by the 50/74 Chief Investigators and 33/46 CTUs who completed the initial surveys. Themes explored include incentives, communication strategies to patients and sites, site training and monitoring and the use of reminders.

35/46 CTUs (76%) participated in the Delphi survey, leading to a ranked list of research priorities, highlighting which are likely to have the broadest impact across a variety of trial contexts.

## Conclusion

A variety of missing data interventions are used depending on trial context but with little evidence to support their use. This project will inform a roadmap for researchers to identify missing data interventions and develop an evidence base.

